# Bacteriophage as an Alternative to Antibiotics Promotes Growth Performance by Regulating Intestinal Inflammation, Intestinal Barrier Function and Gut Microbiota in Weaned Piglets

**DOI:** 10.3389/fvets.2021.623899

**Published:** 2021-01-27

**Authors:** Yongdi Zeng, Zirui Wang, Tiande Zou, Jun Chen, Guanhong Li, Liuzhen Zheng, Shuo Li, Jinming You

**Affiliations:** ^1^Jiangxi Province Key Laboratory of Animal Nutrition, Jiangxi Agricultural University, Nanchang, China; ^2^Jiangxi Province Key Innovation Center of Integration in Production and Education for High-Quality and Safe Livestock and Poultry, Jiangxi Agricultural University, Nanchang, China

**Keywords:** antibiotic-free diet, bacteriophage, diarrhea incidence, growth performance, gut microbiota, intestinal barrier function, intestinal inflammation

## Abstract

This study aimed to investigate the effects of dietary bacteriophage supplementation on growth performance, intestinal morphology, barrier function, and intestinal microbiota of weaned piglets fed antibiotic-free diet. A total of 120 weaned piglets were allotted to four dietary treatments with five pens/treatment and six piglets/pen in a 21-d feeding trial. The control diet was supplemented with 25 mg/kg quinocetone and 11.25 mg/kg aureomycin in the basal diet, while the three treatment diets were supplemented with 200, 400, or 600 mg/kg bacteriophage in the basal diet, respectively. There was no difference for growth performance and all measured indices of serum and intestinal tissues between 200 mg/kg bacteriophage group and the control group with antibiotics (*P* > 0.05). More importantly, compared with the control diet, dietary 400 mg/kg bacteriophage inclusion increased average daily gain and average daily feed intake, and decreased feed/gain ratio and diarrhea incidence of weaned piglets (*P* < 0.05). Also, piglets fed 400 mg/kg bacteriophage had elevated villi height (VH) in jejunum and ileum, reduced crypt depth (CD) in jejunum and ileum, and elevated VH/CD ratio in duodenum, jejunum and ileum (*P* < 0.05). Compared to the control group, piglets fed 400 mg/kg bacteriophage had lower interleukin-1β (IL-1β) and tumor necrosis factor-α (TNF-α), and higher interleukin-10 (IL-10) concentration in serum, and higher secretory immunoglobulin A (sIgA), intestinal trefoil factor (ITF), and tumor growth factor-alpha (TGF-α) content in the ileal mucosa (*P* < 0.05). Besides, dietary addition with 400 mg/kg bacteriophage decreased the D-lactate concentration and diamine oxidase (DAO) activity in serum, and increased the relative mRNA expression of ZO-1, Claudin-1, Occludin, TLR2, TLR4, and TLR9, as well as the relative protein expression of Occludin in the jejunum (*P* < 0.05). However, the growth performance and all analyzed parameters in serum and intestinal tissues were not further improved when piglets fed 600 vs. 400 mg/kg bacteriophage (*P* > 0.05). MiSeq sequencing analysis showed that bacteriophage regulated the microbial composition in caecum digesta, as indicated by higher observed_species, Chao1, and ACE richness indices, as well as changes in the relative abundance of Firmicutes, Bacteroidetes, and Tenericutes (*P* < 0.05). Collectively, 400 mg/kg bacteriophage can be used as an antibiotics alternative for promoting the growth of weaned piglets. The underlying mechanism is associated with a positive effect of bacteriophage on intestinal inflammation, intestinal barrier function and gut microbiota in weaned piglets.

## Introduction

Antibiotics have been widely utilized in pig feed for more than 50 years because of their effectiveness in controlling diseases and promoting growth in swine industry (1). However, the heavy use of antibiotics has been linked to drug-resistant bacteria development and antibiotic resistance dissemination (2, 3). Following the European Union, China banned the use of antibiotics in swine feed in 2020. Under great pressure, pig nutritionists and pig producers urgently seek in-feed antibiotic alternatives for pigs, especially for weaned piglets due to the poor-developed immune system and weaning stress (4, 5).

The bacteriophage is probably the most abundant biological entity on the planet (6). Recently, bacteriophage has acquired practical significance as a means to regulate host immunity and combat pathogenic bacteria in clinical treatments (7–9). It is reported that bacteriophage could regulate innate and adaptive immunity via phagocytosis and cytokine responses (10). Bacteriophage can shape the immunological and metabolic capabilities of the intestine by influencing the stability of the intestinal microbiota (7). Also, studies have found that bacteriophage can regulate the structure of bacterial communities via affecting the parasitic or lytic phase of bacterial cells (11). Bacteriophage may contribute to bacterial colonization and survival in different anatomical sites, especially the favor commensal population to defense disease (12). Dietary addition with bacteriophage increased *Lactobacillus* and *Bifidobacterium* amount, and decreased *Salmonella* and *Coliform* amount in the fecal microbiota of growing pigs (13). More importantly, it's reported that bacteriophage can be used as a growth promoter for growing pigs (13, 14), which provide strong support for the use of bacteriophage in weaned piglets fed antibiotic-free diet.

However, it remains unclear whether bacteriophage could be a potential antibiotic alternative for weaned piglets. Therefore, the objective of this study was to investigate the effects of dietary bacteriophage supplementation on growth performance, intestinal morphology, gut microbiota of weaned piglets fed antibiotic-free diet.

## Materials and Methods

### Ethics Statement

All animal protocols in this study were approved by the Institutional Animal Care and Use Committee of Jiangxi Agricultural University (JXAULL-20190098).

### Animals and Experimental Design

A total of 120 healthy crossbred weaned piglets (Duroc × Landrace × Yorkshire) with an average body weight of 7.35 ± 0.20 kg (25 days of age) were selected, and were randomly assigned by sex and body weight to four dietary treatments with five replicates (pens) per treatment and six piglets per pen. The control diet was supplemented with 25 mg/kg quinocetone and 11.25 mg/kg aureomycin in the basal diet, while the three treatment diets were supplemented with 200, 400, or 600 mg/kg bacteriophage in the basal diet, respectively. The bacteriophage preparation is made of carrier fermented soybean protein, corn flour, and bacteriophage ≥ 1.0 × 10^6^ CFU/g. The basal diet (Table 1) was antibiotic-free, and met or exceeded the required nutrients recommended by the National Research Council (NRC, 2012). The feeding trial lasted for 21 days, during which all piglets had *ad libitum* access to diets and water. The bacteriophage used in this study was a mixture of individual bacteriophage targeting specifically at Salmonella (*Salmonella choleraesuis, Salmonella derby, Salmonella dublin, Salmonella enteritidis, Salmonella gallinarum, Salmonella pullorum, Salmonella typhimurium*), *Escherichia colli* (K88, K99, 987P, F18, F41 and O78), *Clostridium perfringens* (Type A, B, C, D, and E), and *Staphylococcus aureus*. The concentration of individual bacteriophage in the mixture was 10^8^ plaque-forming units per gram (pfu/g).

**Table 1:** The ingredient composition and nutrient level of the basal diet (as-fed basis).

**Item**	**Composition**
**Ingredients (%)**
Expended corn	20.00
Corn	16.70
Expended broken rice	10.00
Wheat flour	10.00
Expanded soybean	8.00
Whey powder	8.00
Peeled soybean meal	4.00
Fish meal	5.00
Soybean meal	4.00
Yeast hydrolysate	4.00
Sucrose	2.50
Glucose	2.50
Soybean oil	1.00
Dicalcium phosphate	1.00
Limestone	0.90
Lysine, 98%	0.60
Methionine	0.27
Threonine	0.23
Salt	0.27
Choline chloride, 60%	0.03
Premix^a^	1.00
Total	100.00
**Nutrient level^b^**
Digestive energy (Mcal/kg)	3.56
Crude protein (%)	18.70
Calcium (%)	0.81
Total phosphorus (%)	0.64
Available phosphorus (%)	0.40
SID lysine (%)	1.23
SID methionine (%)	0.51
SID threonine (%)	0.70
SID tryptophan (%)	0.13
SID valine (%)	0.57

a *The premix provided the following per kg of diet: Fe 170 mg, Mn 40 mg, Zn 110 mg, Co 1.5 mg, Se 0.28 mg, Cu 10 mg, VA 7,000 IU, VD_3_ 2,150 IU, VE 220 mg, VK 12 mg, VB_1_ 2.2 mg, VB_2_ 6 mg, VB_6_ 9 mg, VB_12_ 0.024 mg, biotin 2.5 mg, folic acid 0.9 mg, pantothenic acid 20 mg*.

b *The nutrient levels were calculated values according to Chinese Feed Database (15)*.

### Data and Sample Collection

#### Growth Performance

Piglets were weighed at the beginning and end of the feeding trial, and feed consumption was recorded daily. The average daily gain (ADG), average daily feed intake (ADFI), and feed/gain ratio (F/G) were calculated. All animals were checked for fecal consistency daily using the method of former studies (16). Diarrhea incidence (%) = sum (diarrhea piglet × number of days on diarrhea)/(number of piglets in the pen × number of days of trial) ×100%.

#### Blood Samples

At the end of the feeding trial, five piglets from each diet (one piglet per pen) were randomly selected for sampling blood, intestinal tissues, and caecum degista according to the protocol described by previous methods (17). Blood samples were centrifuged at 3,000 × *g* and 4°C for 10 min to harvest the serum (18). Serum samples were stored at −80°C until further analysis.

#### Intestinal Tissue Samples

After blood was collected, the piglets were euthanized with sodium pentobarbital injection (50 mg/kg body weight), and intestinal tissue samples were collected according to the method in previous study (19). Briefly, intestinal tissues were aseptically sampled from the middle section of duodenum, jejunum, and ileum. The intestinal segments were gently washed with phosphate buffer saline (PBS), and each segment was divided into 2 segments. A 2-cm segment was fixed in 10% formaldehyde-phosphate buffer, and an 18-cm segment was used for collecting mucosal samples. Immediately after collection, mucosal samples were frozen in liquid nitrogen and stored at −80°C until analysis.

#### Caecum Degista Samples

Caecum degista samples were collected in 1.5-mL sterile polypropylene tubes and were immediately frozen in liquid nitrogen, and then kept at −80°C until microbiome analysis.

### Laboratory Analysis

#### Intestinal Morphology

The intestinal morphology was analyzed according to our previous study (20). Briefly, the fixed intestinal samples were dehydrated and then embedded in paraffin. After that, the embedded samples were sectioned and stained with hematoxylin and eosin. The villi height (VH) and crypt depth (CD) were measured using Motic Images Advanced 3.2 software (Motic, Xiamen, China). At least 10-well-oriented intact villis and their associated crypts were analyzed in each intestinal section of each piglet. The CD was divided by VH to calculate the VH/CD ratio.

#### The Concentration of Immunoglobulins and Cytokines in Serum

Levels of immunoglobulin A (IgA), immunoglobulin G (IgG), immunoglobulin M (IgM), interleukin-1β (IL-1β), interleukin-2 (IL-2), interleukin-10 (IL-10), interleukin-12 (IL-12), tumor necrosis factor-α (TNF-α), and interferon-γ (IFN-γ) in serum were determined using enzyme-linked immunosorbent assay (ELISA) kits according to the manufacturer's instructions (Zhongsheng Beikong Biotechnology Co., Ltd, Beijing, China).

#### Ileal Mucosal Barrier Factors

Prior to analysis, the ileal mucosa (0.1 g) was mixed with 0.1 mL physiological saline via tissue homogenate. The tumor growth factor-alpha (TGF-α), intestinal trefoil factor (ITF), major histocompatibility complex II (MHC-II), and secretory immunoglobulin A (sIgA) in ileal mucosa were detected using commercial ELISA kits (Nanjing Jiancheng Bioengineering Institute, Nanjing, China) according to the manufacturer's instructions.

#### D-Lactate Content and Diamine Oxidase (DAO) Activity in Serum

The D-lactate content and DAO activity in serum were determined using commercial kits (Jiancheng Bioengineering Institute, Nanjing, China) according to the protocol described by previous methods (21).

#### Quantitative Real-Time PCR

The quantitative real-time PCR was conducted using the procedures according to our previous studies (22, 23). Briefly, ~100 mg of jejunum mucosa was pulverized with liquid nitrogen. Total RNA was then extracted with 100 mg tissue per milliliter TRIzol (Invitrogen, Carlsbad, CA, USA), and cDNA was synthesized using a reverse transcription kit (Takara Bio, Shiga, Japan). The primers were synthesized by Invitrogen (Shanghai, China) (Table 2). The real-time PCR was performed using a commercial SYBR Green kit (Takara Bio, Shiga, Japan) on a CFX Real-Time PCR Detection System (Bio-Rad, Hercules, CA, USA). The housekeeping GAPDH (glyceraldehyde-3-phosphate dehydrogenase) gene was an internal control to determine the relative expression level of target genes using the 2^−Δ*Δct*^ method (24).

**Table 2:** Specific primers used for real-time quantitative PCR.

**Gene**	**Primer**	**Product size (bp)**	**Accession number**
TLR2	Forward: ACGTATCCATCAATGAACACTGC	146	NM_213761.1
	Reverse: AAGGGTGCAGTCATCAAACTC		
TLR4	Forward: GCAATAGCTTCTCCAGCTTTCC	121	NM_001113039.2
	Reverse: CCCGTCAGTATCAAGGTGGA		
TLR9	Forward: TTCTCTCTACAACCTGGACGC	150	NM_213958.1
	Reverse: TTGAAGGACAGGTTGAGCTTGC		
IL-1β	Forward: GCCCAATTCAGGGACCCTAC	86	NM_214055.1
	Reverse: GGCGGGTTCAGGTACTATGG		
IL-6	Forward: TGGATAAGCTGCAGTCACAG	109	NM_001252429.1
	Reverse: ATTATCCGAATGGCCCTCAG		
TNF-α	Forward: CCAGACCAAGGTCAACCTCC	103	NM_214022.1
	Reverse: TCCCAGGTAGATGGGTTCGT		
ZO-1	Forward: GAGGATGGTCACACCGTGGT	169	XM_021098896.1
	Reverse: GGAGGATGCTGTTGTCTCGG		
Claudin-1	Forward: TCAATACAGGAGGGAAGCCAT	91	NM_001244539.1
	Reverse: ATATTTAAGGACCGCCCTCTCC		
Occludin	Forward: CAGGTGCACCCTCCAGATTG	111	NM_001163647.2
	Reverse: TGGACTTTCAAGAGGCCTGG		
GAPDH	Forward: GAAGGTCGGAGTGAACGGAT	149	NM_001206359.1
	Reverse: CATGGGTAGAATCATACTGGA		

#### Western Blot

The western blot analysis was conducted according to our previous study (23). Briefly, the protein was extracted from the jejunal mucosa, and protein concentration was determined spectrophotometrically using the Protein Quantitative Reagent Kit-BCA Method (Com Win Biotech, Co., Beijing, China). The primary antibodies were as follows: (1) Claudin 1 antibody (1:1,500, ab15098, Abcam, MA, USA), (2) Occludin antibody (1:2,000, 66378-1-lg, Abcam, MA, USA), and (3) ZO-1 antibody (1:1,500, 21773-1-AP, Abcam, MA, USA). β-actin was used as the loading control, and normalization and quantification of the bands were carried out using Quantity-One software.

#### DNA Extraction and MiSeq Sequencing Analysis of Caecum Degista

Genomic DNA was extracted from caecum digesta using a QIAamp DNA Stool Mini Kit (Qiagen Inc., Valencia, CA). The DNA concentration was analyzed using QuantiFluor™-ST (Promega, Madison, WI, USA), and the DNA sample was diluted to 1 ng/μL using sterile water. The bacterial 16S rRNA genes were amplified using the following specific primer pairs (16S V4-V5, 515F-907R): 515F: 5′-GTGCCAGCMGCCGCGG-3′ and 907R: 5′-CCGTCAATTCMTTTRAGTTT-3′. PCR products were mixed in equidensity ratios and purified with a GeneJET Gel Extraction Kit (Thermo Scientific, Waltham, MA, USA). Sequencing of 16S rRNA was performed on an Illumina HiSeq2500 PE250 platform (Illumina Technologies, San Diego, CA, USA) at Zhongke New Life Biotechnology Co., Ltd. (Shanghai, China). Sequencing data were analyzed using the quantitative insights into microbial ecology (QIIME) version 1.9.1. The high-quality sequences were clustered into operational taxonomic units (OTUs) at a similarity level of 97% using UPARSE pipeline (25), and each OTU was annotated with the Greengenes database (13_5 version). The alpha diversity (Chao1, observed_species, ACE, Shannon index, and Simpson index) were calculated by MOTHUR v.1.35.0 (26), and then were statistically analyzed using one-way ANOVA followed by the Duncan multiple comparison method (SPSS 20.0, INC., Chicago, IL, USA). Beta diversity was calculated based on unweighted unifrac distances by QIIME 1.9.1. An unweighted unifrac principal component analysis (PCoA) based on operational taxonomic units (OTUs) was performed to provide an overview of the diversity and composition of caecum microbiota. To determine the statistical differences in beta-diversity of bacterial communities among treatment groups, permutational multivariate analysis of variance (PERMANOVA, Adonis procedure with 999 permutations) in R software (v.3.2.0) was performed to calculate *P*-values. Pairwise Adonis was performed for significant differences among treatments, and the *P*-values were adjusted using the Benjamini–Hochberg correction. The linear discriminant analysis (LDA) effect size (LEfSe) based on the Kruskal-Wallis and Wilcoxon tests was utilized to identify taxa with differentiating relative abundance, and the threshold for the logarithmic LDA score was set at 2.0 for biomarker.

### Statistical Analysis

Statistical analysis were performed using SPSS 22.0 (SPSS, INC., Chicago, IL, USA) and R software (v.3.2.0). Data were tested for normality using the Shapiro-Wilk test before statistical analysis. The data of growth performance, the measured indices in serum and intestinal tissues were analyzed using one-way ANOVA (normality data) Kruskal-Wallis (non-normality data) test followed by the Duncan multiple comparison method. The pen or one killed piglet per pen was used as the experimental unit for all variables. A value of *P* < 0.05 was used for the determination of significant difference, while *P* < 0.10 denoted a tendency.

## Results

### Growth Performance

The effects of dietary bacteriophage supplementation on the growth performance of weaned piglets fed antibiotic-free diet are summarized in Table 3. There was no difference in growth performance between 200 mg/kg bacteriophage group and the control group with antibiotics (*P* > 0.05). However, compared with the control group, dietary 400 mg/kg bacteriophage supplementation increased the final body weight (BW), ADG and ADFI, and decreased F/G and diarrhea incidence of weaned piglets (*P* < 0.05). Interestingly, dietary 600 mg/kg bacteriophage supplementation did not further improve the growth performance of piglets compared to 400 mg/kg bacteriophage group (*P* > 0.05). Dietary bacteriophage supplementation, in both linear and quadratic manners (*P* < 0.05), affected the growth performance of piglets except for F/G (*P* < 0.05).

**Table 3:** Effect of dietary bacteriophage supplementation on growth performance of weaned piglets (*n* = 5)^1^.

**Item**	**Con^2^**	**Treatments^3^**			**SEM**	***P*-value**
		**200 mg/kg bacteriophage**	**400 mg/kg bacteriophage**	**600 mg/kg bacteriophage**		
Initial body weight, kg	7.33	7.33	7.37	7.34	0.04	0.975
Final body weight, kg	11.36^b^	11.53^b^	12.46^a^	12.27^a^	0.16	0.018
Average daily gain, g	213.11^b^	236.79^a, b^	259.45^a^	240.60^a, b^	6.37	0.033
Average daily feed intake, g	339.70^b^	363.95^a, b^	377.89^a^	370.32^a^	5.50	0.044
Feed/gain ratio	1.57^a^	1.56^a^	1.45^b^	1.52^a, b^	0.02	0.014
Diarrhea incidence, %	6.51^a^	6.72^a^	4.71^b^	5.06^b^	0.24	< 0.001

1 *n = 5: there were five replicates (pens) per treatment and six piglets per pen*.

2 *Con: the control diet supplemented with 25 mg/kg quinocetone and 11.25 mg/kg chlortetracycline in the basal diet*.

3 *Treatments: the treatment diets supplemented with 200, 400, or 600 mg/kg bacteriophage in the basal diet*.

a, b *Within a row, values with different letter superscripts differ significantly (P < 0.05)*.

### Intestinal Morphology

**Figure 1:**
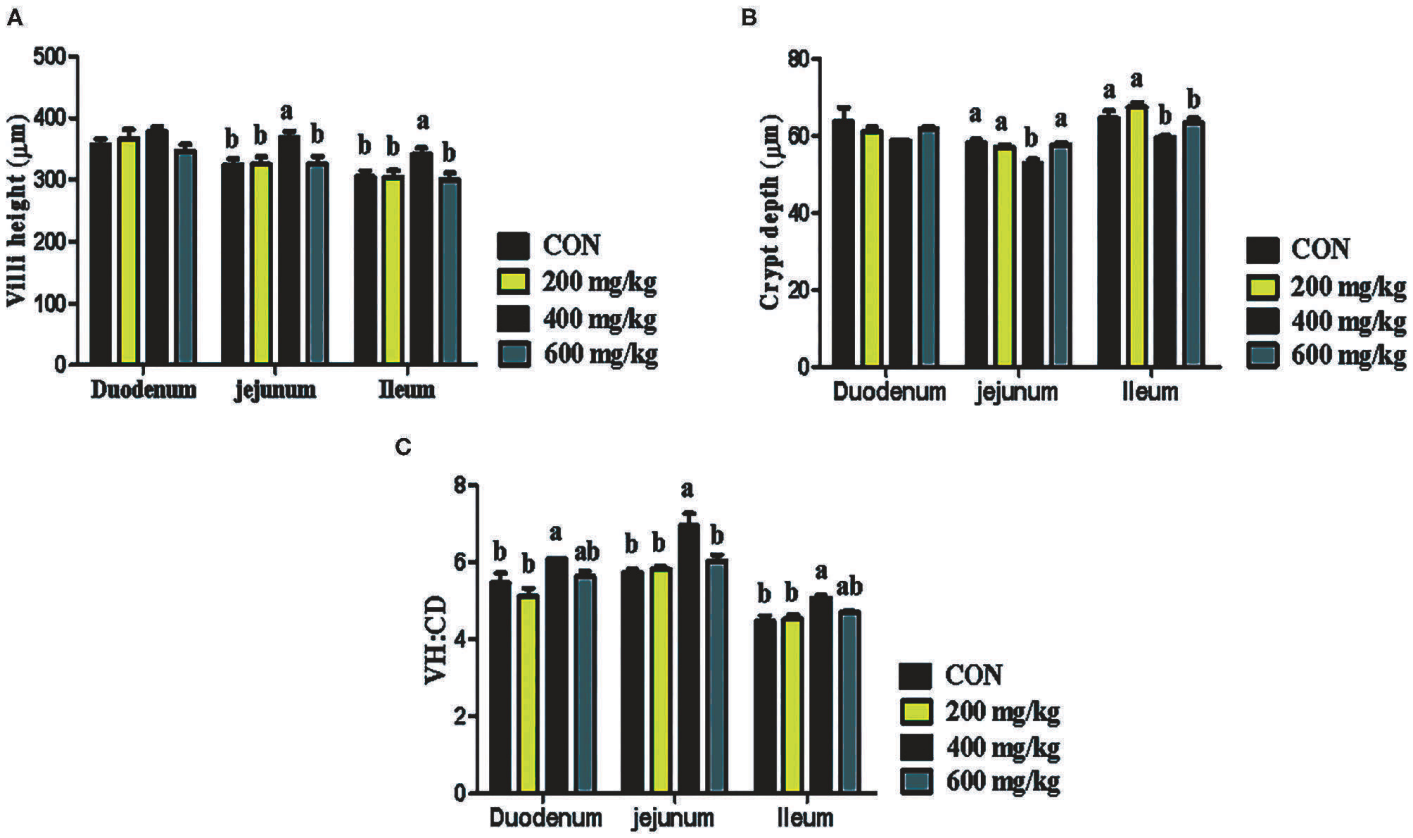
Effects dietary bacteriophage supplementation on intestinal morphology (**A**, Villi height; **B**, Crypt depth; **C**, VH/CD) of weaned piglets fed antibiotic-free diet (*n* = 5). Con: the control diet supplemented with 25 mg/kg quinocetone and 11.25 mg/kg chlortetracycline in the basal diet; Treatments (200, 400, and 600 mg/kg): the treatment diets supplemented with 200, 400, or 600 mg/kg bacteriophage in the basal diet; ^a,b^Different letters above bars indicates significant differences (*P* < 0.05).

As shown in Figure 1, no difference was found for intestinal morphology between 200 mg/kg bacteriophage group and the control group with antibiotics (*P* > 0.05). However, compared to piglets fed the control diet, piglets fed 400 mg/kg bacteriophage diet had elevated VH in jejunum and ileum, reduced CD in jejunum and ileum, and elevated VH/CD in duodenum, jejunum and ileum (*P* < 0.05). Surprisedly, in comparison to 400 mg/kg bacteriophage group, dietary 600 mg/kg bacteriophage supplementation decreased the VH in jejunum and ileum, and increased CD in jejunum and ileum, and decreased VH/CD in the jejunum of piglets (*P* < 0.05).

### Immunoglobulins and Cytokines in Serum

As displayed in Table 4, dietary bacteriophage supplementation did not influence the concentration of IgA, IgG, IgM, IL-2, IL-12, and IFN-γ in the serum of piglets fed antibiotic-free diet (*P* > 0.05). However, piglets fed 400 mg/kg bacteriophage diet had lower IL-1β and TNF-α, and higher IL-10 concentration in serum than piglets fed the control diet (*P* < 0.05). Compared with 400 mg/kg bacteriophage group, dietary 600 mg/kg bacteriophage supplementation decreased serum IL-10 concentration of piglets (*P* < 0.05).

**Table 4:** Effect of dietary bacteriophage supplementation on the concentration of immunoglobulins and cytokines in the serum of weaned piglets fed antibiotic-free diet (*n* = 5)^1^.

**Item**	**Con^2^**	**Treatments^3^**			**SEM**	***P*-value**
		**200 mg/kg bacteriophage**	**400 mg/kg bacteriophage**	**600 mg/kg bacteriophage**		
IgA (g/L)	1.19	1.10	1.23	1.11	0.04	0.585
IgG (g/L)	20.60	20.67	21.12	20.47	0.14	0.405
IgM (g/L)	2.38	2.34	2.41	2.46	0.20	0.160
IL-1β (pg/mL)	28.82^a^	24.64^a, b^	21.44^b^	25.58^a, b^	1.06	0.046
IL-2 (pg/mL)	24.73	29.14	29.03	27.55	0.95	0.335
IL-10 (pg/mL)	16.22^b^	15.32^b^	22.27^a^	16.84^b^	0.84	0.003
IL-12 (pg/mL)	27.73	26.59	24.29	30.57	1.32	0.485
TNF-α (pg/mL)	58.20^a^	58.95^a^	45.93^b^	49.83^a, b^	2.06	0.047
IFN-γ (pg/mL)	48.03	44.36	51.81	46.73	2.80	0.851

*IgA, immunoglobulin A; IgG, immunoglobulin G; IgM, immunoglobulin M; IL-1β, interleukin-1β; IL-2, interleukin-2; IL-10, interleukin-10; IL-12, interleukin-12; TNF-α, tumor necrosis factor-α; IFN-γ, interferon-γ*.

1 *n = 5: there were five replicates (pens) per treatment and six piglets per pen, and one piglet per pen was sampled*.

2 *Con: the control diet supplemented with 25 mg/kg quinocetone and 11.25 mg/kg chlortetracycline in the basal diet*.

3 *Treatments: the treatment diets supplemented with 200, 400, or 600 mg/kg bacteriophage in the basal diet*.

a, b *Within a row, values with different letter superscripts differ significantly (P < 0.05)*.

### Ileal Mucosal Barrier Factors

There was no difference for ileal mucosal barrier factors between 200 mg/kg bacteriophage group and the control group with antibiotics (*P* > 0.05) (Table 5). However, the content of sIgA, ITF, and TGF-α in the ileal mucosa was higher in the 400 mg/kg bacteriophage group than in the control group (*P* < 0.05). No difference was observed for ileal mucosal barrier factors when piglets fed 600 vs. 400 mg/kg bacteriophage diet (*P* > 0.05). A linear or quadratic increase was observed for sIgA and ITF content along with the increased dietary levels of bacteriophage (*P* < 0.05).

**Table 5:** Effect of dietary bacteriophage supplementation on ileal mucosal barrier factors of piglets fed antibiotic-free diet (*n* = 5)^1^.

**Item**	**Con^2^**	**Treatments^3^**			**SEM**	***P*-value**
		**200 mg/kg bacteriophage**	**400 mg/kg bacteriophage**	**600 mg/kg bacteriophage**		
sIgA (μg/mL)	24.12^b^	21.42^b^	69.56^a^	62.35^a^	7.01	0.001
MHC-II (μg/mL)	374.01	374.21	409.31	397.71	16.69	0.880
TGF-α (μg/mL)	905.80^b^	881.61^b^	1141.03^a^	1066.52^a^	38.60	0.013
ITF (μg/mL)	35.89^b^	53.62^a, b^	68.66^a^	68.17^a^	5.09	0.044

*TGF-α, the tumor growth factor-alpha; ITF, intestinal trefoil factor; MHC-II, major histocompatibility complex II; sIgA, secretory immunoglobulin A*.

1 *n = 5: there were five replicates (pens) per treatment and six piglets per pen, and one piglet per pen was sampled*.

2 *Con: the control diet supplemented with 25 mg/kg quinocetone and 11.25 mg/kg chlortetracycline in the basal diet*.

3 *Treatments: the treatment diets supplemented with 200, 400, or 600 mg/kg bacteriophage in the basal diet*.

a, b *Within a row, values with different letter superscripts differ significantly (P < 0.05)*.

### The Relative mRNA Expression of Inflammatory Factors and Toll Like Receptors (TLRs) in the Jejunum

**Figure 2:**
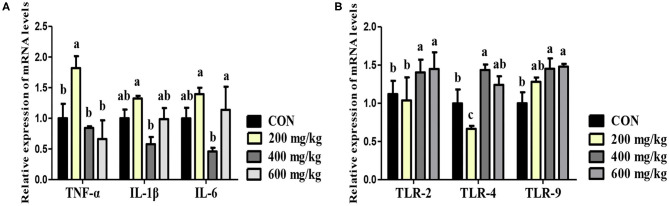
Effects of dietary bacteriophage supplementation on the relative mRNA expression of inflammatory factors **(A)** and TLRs **(B)** in jejunal mucosa of weaned piglets fed antibiotic-free diet (*n* = 5). TNF-α, tumor necrosis factor-α; IL-1β, interleukin-1β; IL-6, interleukin-6; TLR-2, toll like receptor-2; TLR-4, toll like receptor-4; TLR-9, toll like receptor-9. Con: the control diet supplemented with 25 mg/kg quinocetone and 11.25 mg/kg chlortetracycline in the basal diet; Treatments (200, 400, and 600 mg/kg): the treatment diets supplemented with 200, 400, or 600 mg/kg bacteriophage in the basal diet; ^a,b^Different letters above bars indicates significant differences (*P* < 0.05).

Dietary 200 mg/kg bacteriophage supplementation upregulated the relative mRNA expression of TNF-α, and down-regulated the relative mRNA expression of TLR4 in jejunum compared to the control group (*P* < 0.05) (Figure 2). However, the relative mRNA expression of TLR2, TLR4, and TLR9 were upregulated when piglets fed 400 mg/kg bacteriophage in place of the control diet (*P* < 0.05). Besides, the relative mRNA expression of IL-6 (interleukin-6) was upregulated for piglets fed 600 instead of 400 mg/kg bacteriophage diet (*P* < 0.05).

### Intestinal Barrier Function

**Figure 3:**
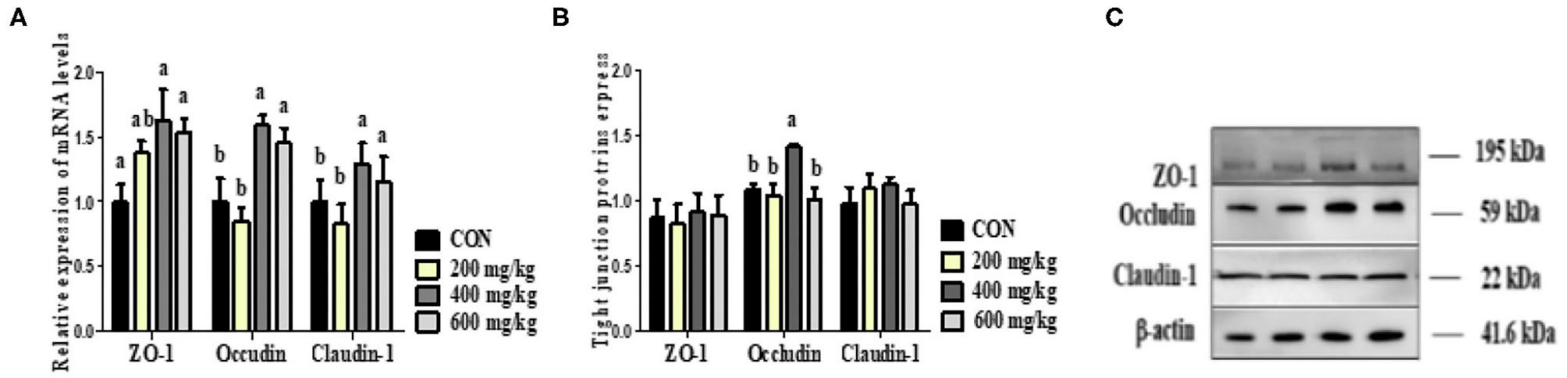
Effects of dietary bacteriophage supplementation on the relative mRNA expression (**A**, *n* = 5) and relative protein expression of tight junction proteins (**B,C**, *n* = 5) in jejunum of weaned piglets fed antibiotic-free diet. Con: the control diet supplemented with 25 mg/kg quinocetone and 11.25 mg/kg chlortetracycline in the basal diet; Treatments (200, 400, and 600 mg/kg): the treatment diets supplemented with 200, 400, or 600 mg/kg bacteriophage in the basal diet; ^a,b^Different letters above bars indicates significant differences (*P* < 0.05).

As presented in Figure 3, the tight junction proteins in jejunum were not differentially expressed in both mRNA level and protein level between 200 mg/kg bacteriophage group and the control group with antibiotics (*P* > 0.05). However, compared to the control group, dietary 400 mg/kg bacteriophage supplementation upregulated the relative mRNA expression of ZO-1, Claudin-1 and Occludin, as well as the relative protein expression of Occludin (*P* < 0.05). No difference was found for tight junction proteins expression when piglets fed 600 vs. 400 mg/kg bacteriophage diet (*P* > 0.05).

As depicted in Table 6, there was no difference for the D-lactate concentration and DAO activity in serum between 200 mg/kg bacteriophage group and the control group with antibiotics (*P* > 0.05). However, the D-lactate concentration and DAO activity were decreased for piglets fed 400 mg/kg bacteriophage diet in place of the control diet (*P* < 0.05). Dietary 600 mg/kg bacteriophage supplementation did not further reduce the D-lactate concentration and DAO activity in comparison to 400 mg/kg bacteriophage group (*P* > 0.05).

**Table 6:** Effect of dietary bacteriophage supplementation on the D-lactate concentration and DAO activity of piglets fed antibiotic-free diet (*n* = 5)^1^.

**Item**	**Con^2^**	**Treatments^3^**			**SEM**	***P*-value**
		**200 mg/kg bacteriophage**	**400 mg/kg bacteriophage**	**600 mg/kg bacteriophage**		
D-lactate (μmol/mL)	11.30^a^	11.85^a^	9.77^b^	10.55^a, b^	0.27	0.018
DAO (U/L)	21.93^a^	23.22^a^	16.25^b^	19.34^a, b^	0.95	0.026

*DAO, diamine oxidase*.

1 *n = 5: there were five replicates (pens) per treatment and six piglets per pen, and one piglet per pen was sampled*.

2 *Con: the control diet supplemented with 25 mg/kg quinocetone and 11.25 mg/kg chlortetracycline in the basal diet*.

3 *Treatments: the treatment diets supplemented with 200, 400, or 600 mg/kg bacteriophage in the basal diet*.

a, b *Within a row, values with different superscripts differ significantly (P < 0.05)*.

### Diversity and Composition of Gut Microbiota

**Figure 4:**
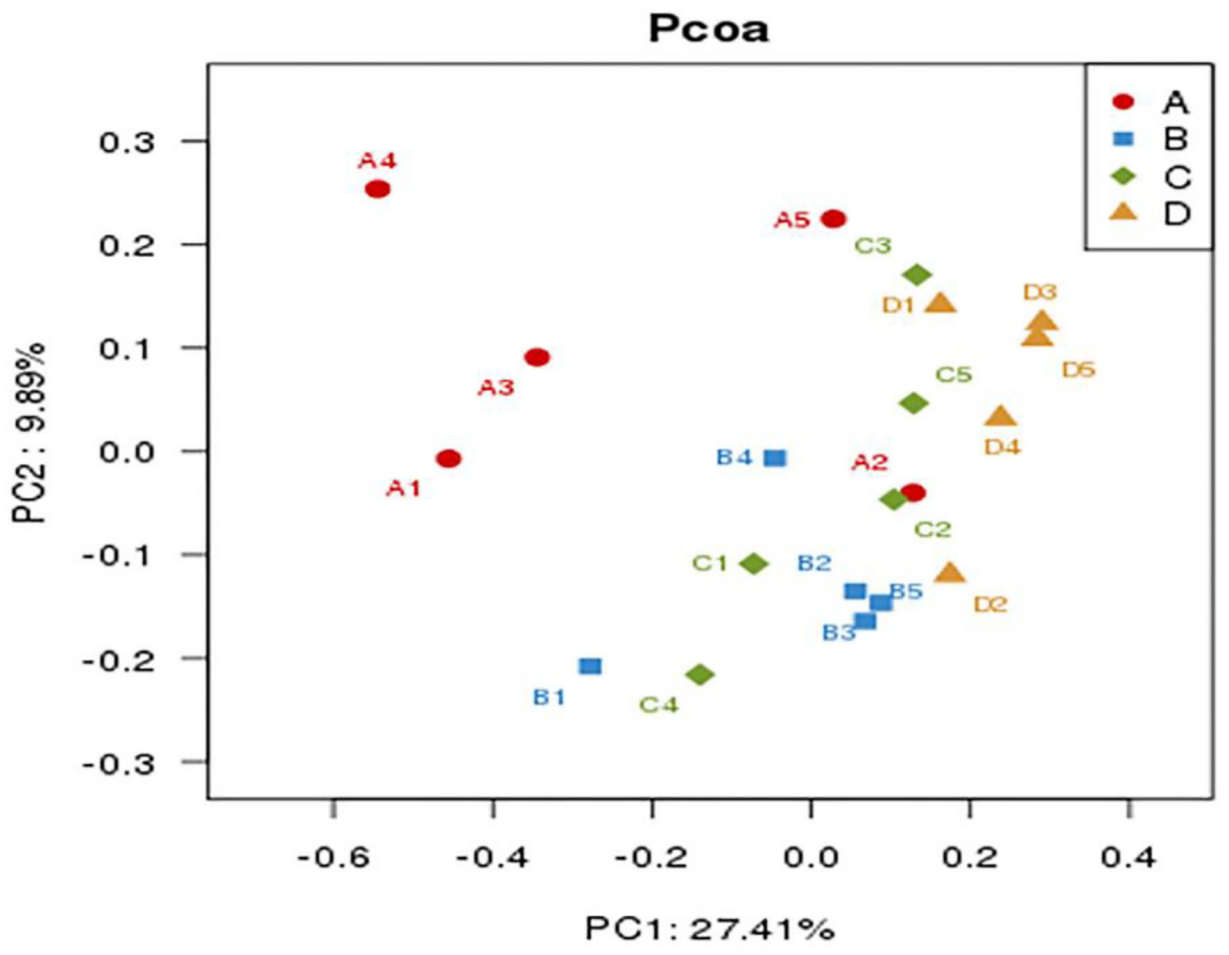
Comparison of caecum microbiota compositions of weaned piglets by principal component analysis (PCoA) (*n* = 5). Group A (A1–A5): the control diet supplemented with 25 mg/kg quinocetone and 11.25 mg/kg chlortetracycline in the basal diet; Group B (B1–B5): 200 mg/kg bacteriophage diet; Group C (C1–C5): 400 mg/kg bacteriophage diet; Group D (D1–D5): 600 mg/kg bacteriophage diet. To determine the statistical differences in beta-diversity of bacterial communities among treatment groups, the PERMANOVA (Adonis procedure with 999 permutations) was performed to calculate *P*-values. There were significant difference between group A and group B (*R*^2^ = 0.191, *P* = 0.017), group A and group C (*R*^2^ = 0.234, *P* = 0.018), group A and group D (*R*^2^ = 0.227, *P* = 0.015), group B and group D (*R*^2^ = 0.155, *P* = 0.042), as well as group C and group D (*R*^2^ = 0.222, *P* = 0.017). No significant difference between group B and group C was found (*R*^2^ = 0.115, *P* = 0.454).

**Figure 5:**
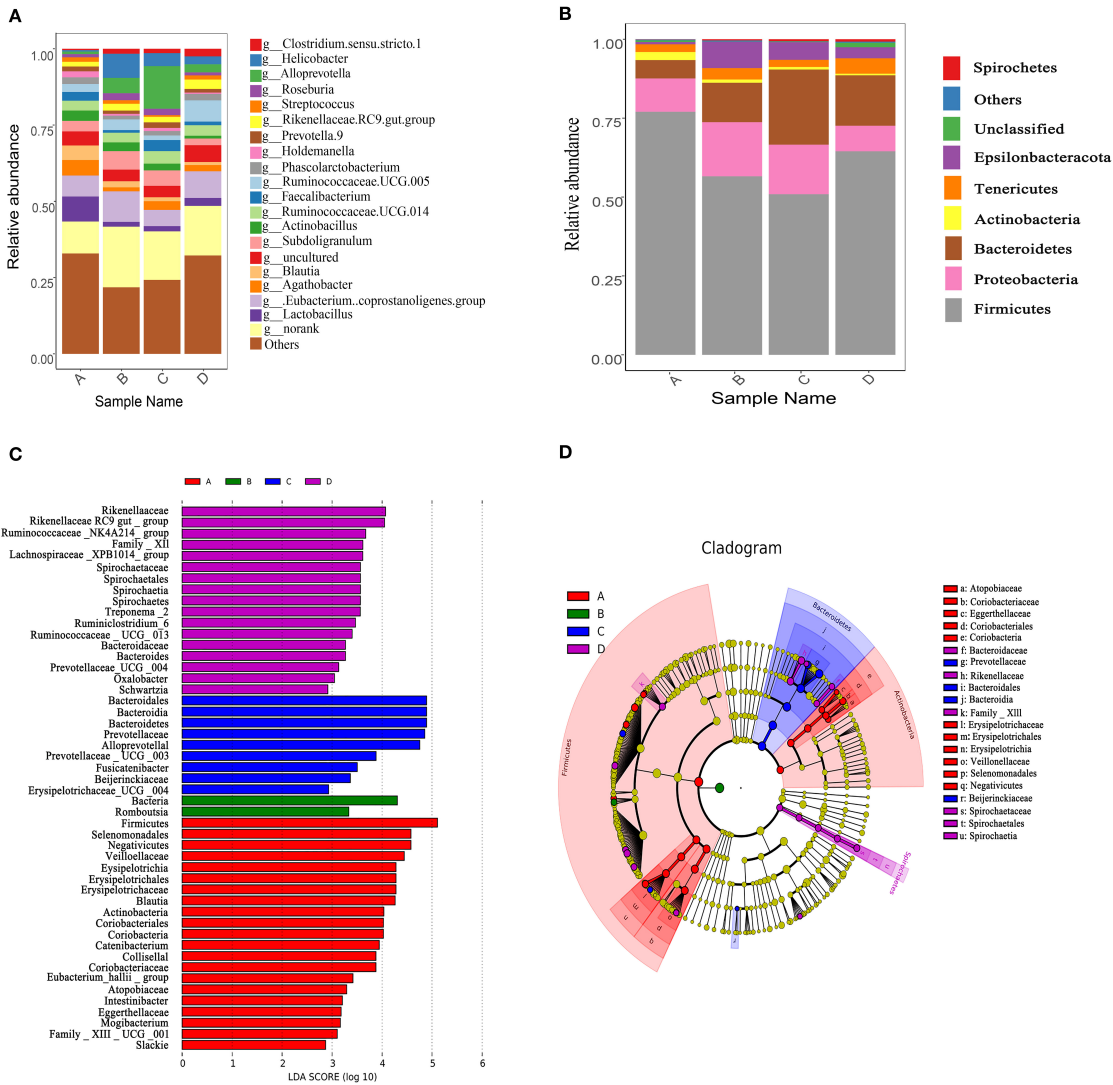
Changes of caecum microbiota compositions of weaned piglets fed with or without dietary bacteriophage (*n* = 5). **(A)** The relative abundance of caecum microbiota composition at the phylum level. **(B)** The relative abundance of caecum microbiota composition at the genus level. **(C)** Cladogram and LDA value distribution histogram. Sample name A: the control diet supplemented with 25 mg/kg quinocetone and 11.25 mg/kg chlortetracycline in the basal diet; Sample name B: 200 mg/kg bacteriophage diet; Sample name C: 400 mg/kg bacteriophage diet; Sample name D: 600 mg/kg bacteriophage diet. The bacterial taxa were significantly differentiated between the bacteriophage group and the control group using linear discriminant analysis coupled with effect size (LEfSe) with the default parameters.

After OTUs were assigned and chimeras were removed, sequencing of 20 samples generated an average of 44,360 ± 1,787 (mean ± standard error) sequences per sample. An average of 2,327 OTUs was identified from these sequences in caecum degista based on 99.98% sequence similarity. The alpha diversity of caecum microbiota is reported in Table 7. Compared with the control group, dietary 400 or 600 mg/kg bacteriophage significantly increased the richness indicators, including observed_species, Chao1, and ACE (*P* < 0.05). Dietary 400 mg/kg bacteriophage supplementation also significantly increased the PD_whole_tree compared to the control group (*P* < 0.05). There were no differences in the diversity index of Shannon and Simpson among experimental groups (*P* > 0.05). As shown in Figure 4, the control group and bacteriophage groups were well separated by PCoA, with principal components PC1 and PC2 explaining 27.4 and 9.89% of the variation, respectively. To determine the statistical differences in beta-diversity of bacterial communities among treatment groups, the PERMANOVA (Adonis procedure with 999 permutations) was performed to calculate *P*-values. There were significant difference between the control group and 200 mg/kg bacteriophage group (*R*^2^ = 0.191, *P* = 0.017), the control group and 400 mg/kg bacteriophage group (*R*^2^ = 0.234, *P* = 0.018), the control group and 600 mg/kg bacteriophage group (*R*^2^ = 0.227, *P* = 0.015), 200 mg/kg bacteriophage group and 600 mg/kg bacteriophage group (*R*^2^ = 0.155, *P* = 0.042), as well as 400 mg/kg bacteriophage group and 600 mg/kg bacteriophage group (*R*^2^ = 0.222, *P* = 0.017). No significant difference between group 200 mg/kg bacteriophage group and 400 mg/kg bacteriophage group was found (*R*^2^ = 0.115, *P* = 0.454). The relative abundance of caecum microbiota is displayed at the phylum level (Figure 5A) and the genus level (Figure 5B). Firmicutes and Bacteroidetes were the primary phyla in caecum microbiota, followed by Proteobacteria and Tenericutes, and Ruminococcaceae, *Eubacterium coprostanoligenes* group, *Alloprevotella*, and *Subdoligranulum* were the major genera. Figure 5C is a cladogram showing the microbiota structure and the predominant bacteria in caecum digesta. There were 28 more taxa in the bacteriophage groups than in the control diet. Specifically, dietary 200 mg/kg bacteriophage supplementation significantly increased the relative abundance of *Bacteria* and *Romboutsia* genera (*P* < 0.05). Compared to the control group, dietary 400 mg/kg bacteriophage supplementation increased six taxa belonging to the Bacteroidetes (*Prevotellaceae* spp. and *Bacteroidales* spp.), two taxa from the Firmicutes (*Erysipelotrichia* spp.), and one taxa from the Proteobacteria phylum (*P* < 0.05). Compared with the control group, dietary 600 mg/kg bacteriophage inclusion increased five taxa from the Firmicutes (*Clostridia* spp. and *Veillonellaceae* spp.), five taxa from the Bacteroidetes (*Bacteroidales* spp. and *Rikenellaceae* spp.), five taxa from the *Spirochaetes* (*Spirochaetales* spp. and *Treponema_2* spp.), and one taxa from the Proteobacteria phylum (*P* < 0.05).

**Table 7:** Effect of dietary bacteriophage supplementation on the alpha diversity of caecum microbiota in weaned piglets fed antibiotic-free diet (*n* = 5)^1^.

**Item**	**Con^2^**	**Treatments^3^**			**SEM**	***P*-value**
		**200 mg/kg bacteriophage**	**400 mg/kg bacteriophage**	**600 mg/kg bacteriophage**		
Observed_species	879.40^c^	982.60^c, b^	1,052.60^a, b^	1,171.75^a^	33.86	0.011
Shannon	4.64	4.53	4.92	5.22	0.11	0.140
Simpson	0.03	0.05	0.03	0.02	0.01	0.228
Chao1	1,242.80^b^	1,388.76^a, b^	1,533.20^a^	1,593.13^a^	45.15	0.011
ACE	1,168.28^b^	1,326.72^a, b^	1,380.85^a^	1,455.43^a^	36.52	0.030
PD_whole_tree	12.70^b^	13.86^a, b^	15.06^a^	14.50^a, b^	0.34	0.045

1 *n = 5: there were five replicates (pens) per treatment and six piglets per pen, and one piglet per pen was sampled*.

2 *Con: the control diet supplemented with 25 mg/kg quinocetone and 11.25 mg/kg chlortetracycline in the basal diet*.

3 *Treatments: the treatment diets supplemented with 200, 400, or 600 mg/kg bacteriophage in the basal diet*.

a, b, c *Within a row, values with different superscripts differ significantly (P < 0.05)*.

## Discussion

The objective of this study was to evaluate the effects of dietary bacteriophage supplementation on growth performance, intestinal morphology, barrier function, and gut microbiota of weaned piglets fed antibiotic-free diet. In the present study, we used the antibiotic group as the control based on the following explanation: The original intention and key focus is to find out an antibiotic alternative for weaned piglets. Because the in-feed antibiotic is widely-recognized in swine industry, we selected the antibiotic group as the control group to find out an optimal dosage of bacteriophage to catch up the additive effect of widely-recognized standard control, and replace it.

It is demonstrated that bacteriophage can be used as a growth promoter for growing pigs (13, 14). One study found that dietary supplementation with anti-*Salmonella Typhimurium* bacteriophage increased ADG and G/F of *Salmonella*-challenged growing pigs (14). In another experiment also found that dietary bacteriophage supplementation improved ADG and ADFI of growing pigs, which provide strong support for the use of bacteriophage in weaned piglets fed antibiotic-free diet (13). In the present study, there was no difference for growth performance between 200 mg/kg bacteriophage group and the control group with antibiotics, which indicates 200 mg/kg bacteriophage can be used as an alternative of antibiotics for promoting the growth of weaned piglets. More importantly, dietary 400 mg/kg bacteriophage supplementation increased the final BW, ADG and ADFI, and decreased F/G and diarrhea incidence of weaned piglets compared to the control group (*P* < 0.05). That is to say, dietary 400 mg/kg bacteriophage supplementation greatly improved growth performance in comparison to the antibiotic diet. However, no difference was observed for growth performance when piglets fed 600 vs. 400 mg/kg bacteriophage diet (*P* > 0.05). Our results suggest that bacteriophage is a potentially effective alternative to in-feed antibiotics for promoting the growth of weaned piglets, and 400 mg/kg bacteriophage is recommended for weaned piglets fed antibiotic-free diet.

To understand the underlying mechanism about the growth-promoting effect of bacteriophage on weaned piglets fed antibiotic-free diet, the serum, intestinal tissues and digesta were collected and measured. Intestinal morphology is a vital indicator of gut health (27), as well as the digestive and absorptive capacity of the intestine (28). Weaning is reported to induce significant changes in intestinal morphology, including decreased villi height and increased crypt depth, which generally leads to diarrhea of weaned piglets (29–31). In the present experiment, compared to pigs fed the control diet, piglets fed 400 mg/kg bacteriophage diet had elevated VH in jejunum and ileum, reduced CD in jejunum and ileum, and elevated VH/CD in duodenum, jejunum and ileum (*P* < 0.05), which confirmed the improved diarrhea incidence of weaned piglets fed 400 mg/kg bacteriophage diet. In agreement with our results, Monsur et al. (32) found that bacteriophage was as capable as tetracycline in alleviating diarrhea of patients without any apparent toxic effect. Also, previous studies on bacteriophage aimed at treating pathogenic *E. coli* in lambs, calves, and pigs have achieved promising results (33). Besides, better intestinal morphology is associated with better intestinal digestive and absorptive capacity, which is confirmed by previous study (34). Meanwhile, studies have reported that 0.025 or 0.050% anti-*Salmonella* bacteriophage improved dry matter, energy, and nitrogen digestibility of growing pigs (34).

As the biggest immune organ, the intestine can secrete bioactive substances to defend against foreign antigens, toxins, and macromolecules (30). It was reported that bacteriophage is a modulator of immune responses in both specific and non-specific immune manners (7). In the present study, 400 mg/kg bacteriophage enhanced the immune capacity of weaned piglets, as indicated by the increased content of sIgA, TGF-α and ITF in the ileal mucosa. Consistent with improved immunity, 400 mg/kg bacteriophage increased the content of anti-inflammatory factor (IL-10), and decreased the content of pro-inflammatory factor (IL-1β) and TNF-α in the serum of piglets. In line with our results, Tothova et al. (35) reported that bacteriophage therapy decreased the expression of pro-inflammatory cytokines of mice with urinary tract infections. Similarly, one study found that bacteriophage therapy inhibited inflammatory cytokine production induced by *Klebsiella pneumonia*-mediated liver abscess and bacteremia in mice (36). Bacteriophage was also reported to inhibit tissue expression of inflammatory factors in mice (37). When the body is invaded by foreign pathogens, TLR2, TLR4, TLR9, and other receptors are rapidly triggered (38). The activated TLRs could provide a message about the bacterial census in the intestine, and trigger the expression of secretory anti-microbial proteins in order to maintain mucosal surface-related bacterial populations at homeostatic levels (39). The present study suggests that dietary bacteriophage supplementation promotes the mRNA expression of TLR2, TLR4, and TLR9 in the jejunum mucosa of weaned piglets, which indicates that bacteriophage activates the immune system by regulating the TLR-mediated inflammatory response of weaned piglets fed antibiotic-free diet.

The intestinal barrier integrity is essential for maintaining the normal physiological functions of the epithelial cells and blocking pathogenic bacteria that may induce inflammation. Intestinal barrier damage increases epithelial permeability (40). Tight junction proteins are the principal determinants of epithelial and endothelial paracellular barrier functions (41). A layer of epithelial cells is held together by tight junction proteins (such as Claudin 1, Occludin, and ZO-1) to maintain the intestinal barrier integrity (42). The D-lactate concentration and DAO activity are also used as indicators of intestinal barrier function (43–45). Recent studies have showed that bacteriophages contribute to the intestinal health and diarrhea improvement of weanling piglets, which may be the reflection of improved intestinal barrier function by bacteriophages supplementation (46, 47). Lee et al. (46) reported that dietary supplementation with 0.10% bacteriophage cocktail enhanced intestinal health of weanling piglets, as indicated by the improved fecal score, intestinal morphology and intestinal absorption. Hosseindoust et al. (47) also found that dietary supplementation with 0.10% bacteriophage cocktail resulted in better growth performance, digestibility, and gut development of weanling piglets, and the additive effect of bacteriophage cocktail is comparable to zinc oxide supplementation. To the best of our knowledge, no reports are available about the impacts of bacteriophage on the intestinal barrier function of pigs. In the present study, compared to the control group, dietary 400 mg/kg bacteriophage supplementation upregulated the relative mRNA expression of ZO-1, Claudin-1 and Occludin, as well as the relative protein expression of Occludin (*P* < 0.05), which was complemented by reduced serum D-lactate concentration and DAO activity. Our results suggest that 400 mg/kg bacteriophage can protect the intestinal barrier integrity of weaned piglets fed antibiotic-free diet.

Intestinal microbiota dysfunction is associated with impaired intestinal mucosal barrier and compromised growth performance in piglets (3). Diversity is known to improve the stability and performance of communities (48). Studies have shown that in high risks populations such as premature infants, children receiving antibiotics, and traveler's diarrhea in adults, the potential benefits of modifying the composition of the intestinal microbiome for therapeutic effects are clear (49). In the current study, numerous OTUs and species richness (Chao1 and observe_species) in caecum microbiota were increased in piglets fed a bacteriophage-supplemented diet, which illuminates a regulatory effect of bacteriophage on the intestinal microbiota. It's most likely that bacteriophage regulates the microbial population with substantial turnover, thus significantly affecting bacterial abundance/diversity and metabolism in the gut (50). The alpha-diversity results revealed that dietary bacteriophage supplementation remarkably increased the richness of the caecum microbiota. As for the effects of bacteriophage dosage on bacterial composition, the Observed_species index was found to be increased by 600 vs. 200 mg/kg bacteriophage supplementation. Also, the relative abundance of *Alloprevotell* was increased, while the relative abundance of *Rikenellaceae RC9 gut group* was decreased in the 400 vs. 200 mg/kg bacteriophage group (Supplementary Table 1). It was reported that, with the increase of dietary bacteriophage levels, the number of *Clostridium* spp. and *Coliforms* were decreased, but the number of *Bifidobacterium* spp., *Lactobacillus* spp. and total anaerobic bacteria were increased in feces of pigs (13). Consistent with our results, Bao et al. (51) reported that phage treatment increased alpha-diversity of fecal microbiota of mice, such as Chao1 and Shannon index. No significant changes in phage-targeted bacteria were detected in the present study. The best explanation of the increased alpha-diversity by bacteriophage is that phages appear to exert significant selective pressure on pathogenic bacteria while at the same time contributing to bacterial diversity (i.e., “killing the winner”) (52). The increase of microbial abundance is associated to enhancing the stability of the ecosystem and resistance to pathogen invasion (53). Thence, the increased diversity in the intestinal microbiota by bacteriophage most likely contribute to the improved intestinal mucosa immune system. The PCoA results suggest that bacterial composition varied among treatments, and the composition was closer in the bacteriophage group than in the control group. Bacteriophage may contribute to bacterial colonization and survival in different anatomical sites, especially the favor commensal population to defense disease. Firmicutes, Bacteroidetes, Proteobacteria, and Epsilonbacteraeota were the predominant phyla in caecum microbiota. Consistent with previous studies in pigs (54), the ratio of Firmicutes to *Bacteroides* was positively correlated with weight gain, and *Firmicutes* improved the energy utilization of diet (55, 56). However, the results of this experiment showed that the ratio of *Firmicutes* to *Bacteroides* in each bacteriophage group was lower than that in the control group, which may be related to the rapid replication and function of bacteriophage in piglets. As a result, the ratio of Firmicutes to *Bacteroides* was lower than that of the antibiotic group in the late stage of treatment. The results of previous studies have shown that with the age of piglets, the relative abundance of *Firmicutes* and *Proteobacteria* significant declined, and the relative abundance of *Bacteroides* and Fibrobacteres significant increased (57). This is consistent with the results of this study, the content of *Bacteroidetes* in the bacteriophage group was significantly higher than that of the antibiotic group in the late treatment period. The previous study demonstrated a relationship between host weight and increased abundance of rumen *Bacillus* and *Bacteroides* (58), which was confirmed by studies on the intestinal microbiota of mice and humans (59, 60). The present study suggests that gut microbiota regulation may be another underlying mechanism of the growth-promoting of bacteriophage for the weaned piglet. Further researches are needed to explore the effects of bacteriophage on the targeted bacteria in the intestine of piglets.

## Conclusion

In conclusion, bacteriophage can be used as an in-feed antibiotics alternative for promoting growth, and 400 mg/kg bacteriophage is recommended for weaned piglets fed antibiotic-free diet. The underlying mechanism is associated with a positive effect of bacteriophage on intestinal inflammation, intestinal barrier function and gut microbiota in weaned piglets.

## Data Availability Statement

The 16S sequencing data used in this study has been deposited to the Sequence Read Archive (SRA) of National Center for Biotechnology Information (NCBI), and the SRA accession number is PRJNA682292.

## Ethics Statement

The animal study was reviewed and approved by Institutional Animal Care and Use Committee of Jiangxi Agricultural University.

## Author Contributions

JY and GL conceived and designed the whole trial. YZ, ZW, and SL conducted the animal trial. TZ, JC, and LZ conducted the laboratory analysis. YZ, ZW, and JY wrote the manuscript. All authors agree to be accountable for the content of the work.

## Conflict of Interest

The authors declare that the research was conducted in the absence of any commercial or financial relationships that could be construed as a potential conflict of interest.
