# Supplementary material for: Bacteriophage as an Alternative to Antibiotics Promotes Growth Performance by Regulating Intestinal Inflammation, Intestinal Barrier Function and Gut Microbiota in Weaned Piglets
Source: Front Vet Sci. 2021 Jan 27;8:623899. doi: 10.3389/fvets.2021.623899 (PMC7874526; doi:10.3389/fvets.2021.623899)
Supplement: Supplementary file 1 [file Table_1.docx]

**Supplementary Materials**

**Table S1.** The relative abundance of caecum microbiota composition at the genus level (n=5) ^1^.

| Item | Con^2^ | Treatments^3^ | | | SEM | *P*-value |
| --- | --- | --- | --- | --- | --- | --- |
|  |  | 200 mg/kg bacteriophage | 400 mg/kg bacteriophage | 600 mg/kg bacteriophage |  |  |
| *Rikenellaceae RC9 gut group* | 0.0043^b^ | 0.0162^a^ | 0.0143^b^ | 0.0248^a^ | 0.0026 | 0.026 |
| *Helicobacter* | 0.0047 | 0.0792 | 0.0425 | 0.0258 | 0.0164 | 0.460 |
| *Alloprevotella* | 0.0083^b^ | 0.0502^b^ | 0.1396^a^ | 0.0266^b^ | 0.0154 | 0.003 |
| *Roseburia* | 0.0105 | 0.0230 | 0.0211 | 0.0099 | 0.0035 | 0.422 |
| *Streptococcus* | 0.0148 | 0.0116 | 0.0056 | 0.0136 | 0.0030 | 0.752 |
| *Clostridium sensu stricto 1* | 0.0156 | 0.0224 | 0.0179 | 0.0309 | 0.0040 | 0.574 |
| *Prevotella 9* | 0.0157 | 0.0106 | 0.0184 | 0.0118 | 0.0034 | 0.867 |
| *Holdemanella* | 0.0195^a^ | 0.0060^b^ | 0.0102^ab^ | 0.0042^b^ | 0.0024 | 0.086 |
| Phascolarctobacterium | 0.0222 | 0.0123 | 0.0145 | 0.0213 | 0.0036 | 0.743 |
| *Ruminococcaceae UCG-005* | 0.0259^b^ | 0.0348^b^ | 0.0148^b^ | 0.0695^a^ | 0.0072 | 0.030 |
| *Faecalibacterium* | 0.0286 | 0.0088 | 0.0365 | 0.0120 | 0.0053 | 0.199 |
| *Ruminococcaceae UCG-014* | 0.0323 | 0.0316 | 0.0409 | 0.0344 | 0.0042 | 0.877 |
| *Actinobacillus* | 0.0339 | 0.0287 | 0.0222 | 0.0092 | 0.0067 | 0.622 |
| *Subdoligranulum* | 0.0343 | 0.0606 | 0.0504 | 0.0220 | 0.0062 | 0.112 |
| uncultured^4^ | 0.0462 | 0.0381 | 0.0375 | 0.0551 | 0.0030 | 0.120 |
| *Blautia* | 0.0477^a^ | 0.0201^b^ | 0.0128^b^ | 0.0098^b^ | 0.0046 | 0.003 |
| *Agathobacter* | 0.0509 | 0.0131 | 0.0286 | 0.0207 | 0.0067 | 0.227 |
| *Eubacterium coprostanoligenes group* | 0.0686 | 0.1001 | 0.0531 | 0.0872 | 0.0087 | 0.248 |
| *Lactobacillus* | 0.0821^a^ | 0.0158^b^ | 0.0172^b^ | 0.0261^b^ | 0.0095 | 0.023 |
| norank^5^ | 0.1046 | 0.1987 | 0.1593 | 0.1626 | 0.0205 | 0.473 |
| Others | 0.3293^a^ | 0.2182^b^ | 0.2424^b^ | 0.3226^a^ | 0.0167 | 0.020 |

^1^n=5: there were 5 replicates (pens) per treatment and 6 piglets per pen, and one piglet per pen was sampled; ^2^Con: the control diet supplemented with 25 mg/kg quinocetone and 11.25 mg/kg chlortetracycline in the basal diet; ^3^Treatments: the treatment diets supplemented with 200, 400, or 600 mg/kg bacteriophage in the basal diet; ^4^“uncultured” in the taxonomy notes means that the bacteria have not been cultured purely; ^5^“norank” means there is no clear information on classification or category name at the taxonomic level. ^a,b^Within a row, values with different superscripts differ significantly (*P*<0.05). Data were tested for normality using the Shapiro-Wilk test before statistical analysis. Data were analyzed using one-way ANOVA (normality data) Kruskal-Wallis (non-normality data) test followed by the Duncan multiple comparison method.
